# Numerical Analysis of the Correlation between Arc Plasma Fluctuation and Nanoparticle Growth–Transport under Atmospheric Pressure

**DOI:** 10.3390/nano9121736

**Published:** 2019-12-06

**Authors:** Masaya Shigeta, Manabu Tanaka, Emanuele Ghedini

**Affiliations:** 1 Joining and Welding Research Institute, Osaka University, Osaka 567-0047, Japan; tanaka@jwri.osaka-u.ac.jp; 2 Alma Mater Studiorum, University of Bologna, 40126 Bologna, Italy; emanuele.ghedini@unibo.it

**Keywords:** arc plasma, fluid dynamics, nanoparticles, simulation, transport phenomena

## Abstract

A time-dependent two-dimensional (2D) axisymmetric simulation was conducted for arc plasma with dynamically fluctuating fluid generating iron nanoparticles in a direct-current discharge condition. The nonequilibrium process of simultaneous growth and transport of nanoparticles is simulated using a simple model with a low computational cost. To ascertain fluid dynamic instability and steep gradients in plasma temperature and particle distributions, a highly accurate method is adopted for computation. The core region of the arc plasma is almost stationary, whereas the fringe fluctuates because of fluid dynamic instability between the arc plasma and the shielding gas. In the downstream region, the vapor molecules decrease by condensation. The nanoparticles decrease by coagulation. These results suggest that both of the simultaneous processes make important contributions to particle growth. The fluctuation of nanoparticle number density in a distant region exhibits stronger correlation with the temperature fluctuation at the plasma fringe. The correlation analysis results suggest that the distribution of growing nanoparticles distant from the arc plasma can be controlled via control of temperature fluctuation at the arc plasma fringe.

## 1. Introduction

**Figure 1:**
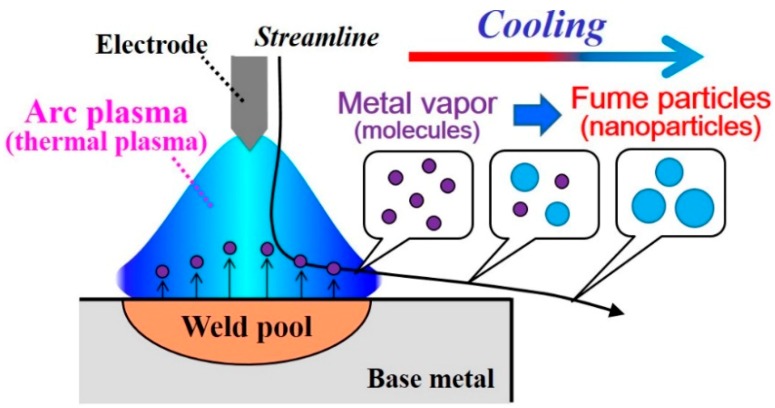
Schematic illustration of nanoparticle production by arc plasma. Metal vapor is generated by the high-temperature plasma. Vapor molecules are transported outside the plasma and therein form nanoparticles.

During arc welding processes operated under atmospheric pressure, smoke released from the welding region is often observed. This smoke, so-called “welding fumes”, is composed of aggregates of ultrafine particles with diameters ranging from subnanometers to a few hundred nanometers [1,2]. Fume nanoparticles can cause health problems for welders when inhaled [3]. Reduction of fume nanoparticles is therefore an important arc welding issue. From a different perspective, arc welding can be regarded as a nanoparticle production method using thermal plasma, as presented in Figure 1. Thermal plasma has very high interior temperatures of more than 10,000 K and high cooling rates at its fringe. These features have led to one-step fabrication of nanoparticles at high rates [4]. In fact, nanoparticles have been in high demand [5,6], as are nanowires [7,8], for various applications. Better techniques must be developed to control nanoparticle production rate and size by understanding the mechanisms of nanoparticle growth and transport processes.

Using arc plasma, Tanaka and Watanabe [9] conducted an experimental investigation of Sn–Ag alloy nanoparticle formation in a system. Other experiments related to fume generation in arc welding have been conducted mostly for purposes of detoxification and reduction of fume particles. Jeskins et al. [10] used energy dispersive X-ray analysis for specific investigation of the composition of nanoparticles generated through arc welding of several types. Berlinger et al. [1] used a scanning mobility particle sizer or a transmission electron microscope to measure the size distribution of nanoparticles generated in various welding processes. Those measurements indicated that almost all nanoparticles had diameters of less than a few tens of nanometers. Using a transmission electron microscope, Carpenter et al. [11] observed nanoparticles generated under different welding conditions. They clarified that most nanoparticles were spherical single crystals smaller than 100 nm. However, due to technological limitations, observing and measuring every process of simultaneous growth and transport of nanoparticles around arc plasma is difficult.

Theoretical modeling and numerical studies are effective approaches, as demonstrated by other thermal plasma systems with nanopowder formation [12,13,14,15,16,17,18,19,20,21]. For an arc plasma process, Tashiro et al. [22] conducted a numerical calculation based on an oversimplification that small particles do not collide and that only large particles collide and form agglomerates in a two-dimensional space. However, neither the yield nor size distribution of particles was obtained in practical space and time scales. Sanibondi [23] also modeled nanoparticle formation involving iron oxidation reactions and agglomeration using a stochastic approach under a simple linear cooling condition. Shigeta et al. [24] used a different model capable of expressing a particle size distribution with any shape and capable of calculating its transient behavior because of the collective growth of particles through homogeneous nucleation, heterogeneous condensation, and coagulation among particles. They were able to do so even if particles had widely various diameters of subnanometers to a few hundred nanometers. Furthermore, they improved their model to calculate the collective growth of iron nanoparticles with charging caused by collision with ions and electrons from the arc plasma [25]. It is noteworthy that, because those models required large computational resources, the collective growth was clarified for only simple temperature histories along some typical one-dimensional (1D) streamlines around the arc plasma. Boselli et al. [26] conducted a two-dimensional (2D) axisymmetric simulation using the method of moments (MOM) [27] for iron nanoparticle generation near arc plasma in a pulse discharge condition. That effort was possible because MOM can calculate the simultaneous growth and transport of nanoparticles with a lower computational cost [13,14]. Nevertheless, its mathematical formulation might be too intricate for engineering use. To avoid this shortcoming, a more sophisticated model with a lower computational cost was developed for nanoparticle generation with a thermal plasma jet [18,21,28]. This study also adopted the model for arc plasma in a direct-current discharge condition.

As described above, some studies have examined nanoparticle generation around arc plasma [22,23,24,25,26]. However, no report in the relevant literature describes a study of the correlation between nanoparticle growth–transport and arc plasma fluctuation caused by fluid dynamic instability. Fluid dynamic fluctuation is a characteristic of fluid flow even for plasma. Therefore, it affects nanoparticle growth and transport. This study clarifies the correlation between those two physics using a time-dependent 2D axisymmetric simulation. The present growth–transport model expresses nonequilibrium processes of collective particle growths by homogeneous nucleation, heterogeneous condensation, and coagulation among particles, as well as transports by convection and diffusion of nanoparticles. To capture fluid dynamic instability and steep gradients in plasma temperature and particle distributions, a highly accurate method [29] is adopted for computation. The present simulation treats a large domain specifically to assess the correlation between the arc plasma fluctuation and the nanoparticle distribution in a far downstream region.

## 2. Model Description

**Figure 2:**
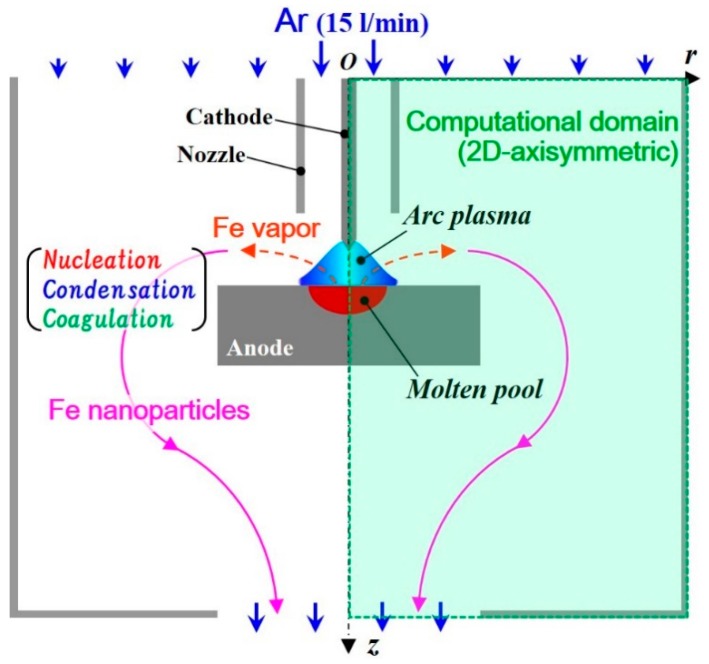
Computational domain of an arc plasma system for nanoparticle production and collection. Iron vapor molecules are emitted from a molten pool and transported outside the arc plasma. Therein, the molecules form nanoparticles through growth processes of nucleation, condensation, and coagulation.

Figure 2 portrays the present computational domain of an arc plasma system for nanoparticle production and collection described in the 2D axisymmetric coordinate system with axial position *z* and radial position *r*. Table 1 presents the conditions of the arc plasma discharge. Arc plasma was generated between a cathode and an anode with a 5.0 mm gap under atmospheric pressure. The cathode was a 3.2 mm diameter, 25.0 mm long wire of tungsten containing 5.0 wt % of La_2_O_3_ with a 60-degree tip angle. The anode was an iron plate with 40.0 mm diameter and 10.0 mm thickness. Argon shielding gas was injected at 15.0 L/min from an iron nozzle with a 12.2 mm inner diameter, 1.0 mm thickness, and 20.0 mm length. The current and voltage were set as 150.0 A and 10.5 V, respectively. The ground voltage was set at the anode bottom. To collect nanoparticles effectually, this arc plasma discharge part was covered by an iron case with a 100.0 mm diameter, 80.0 mm length, and 40.0 mm outlet diameter. A slow argon flow was also supplied from the top at 0.25 m/s to prevent metal vapor and nanoparticles from going out upward and to collect all the nanoparticles at the bottom outlet. It is noteworthy that this slow flow did not strongly affect the arc plasma. The temperature of this case was fixed at 300.0 K.

During arc plasma discharge, high-temperature metal vapor is always generated from the molten pool surface of the anode [30]. The metal vapor is transported with a shielding gas flow to the outside of the arc plasma, where the metal vapor is cooled rapidly. With decreasing temperature, the saturation pressure of the metal vapor decreases; then, the metal vapor pressure exceeds the saturation vapor pressure. This supersaturated state engenders phase change from the vapor to nanoparticles through homogeneous nucleation, heterogeneous condensation, and coagulation. Simultaneously, convection and diffusion transport the nanoparticles.

The present system can be described mathematically based on several assumptions: (a) the entire fluid region including plasma and non-ionized gas is in a local thermodynamic equilibrium (LTE) state; (b) the plasma is optically thin; (c) the convection in the molten pool is ignored; (d) the mass loss at the molten pool surface caused by evaporation is negligible; (e) nanoparticles are liquid spherical particles because of melting point depression effects on a nanometer scale [31]; (f) electric charge effects are ignored; (g) particle temperature is equal to the fluid temperature; and (h) metal vapor is treated as an ideal gas.

The flow, temperature, and electromagnetic fields are obtained by solving the following governing equations:

(1) $$\frac{\partial \rho }{\partial t}+\frac{\partial }{\partial z}\left(\rho u_{z}\right)+\frac{1}{r}\frac{\partial }{\partial r}\left(r\rho u_{r}\right)=S_{m},$$

(2) $$\begin{array}{cc} \frac{\partial }{\partial t}\left(\rho u_{z}\right)+\frac{\partial }{\partial z}\left(\rho u_{z}u_{z}\right)+\frac{1}{r}\frac{\partial }{\partial r}\left(r\rho u_{z}u_{r}\right)= & -\frac{\partial P}{\partial z}+2\frac{\partial }{\partial z}\left(\eta \frac{\partial u_{z}}{\partial z}\right)+\frac{1}{r}\frac{\partial }{\partial r}\left[r\eta \left(\frac{\partial u_{z}}{\partial r}+\frac{\partial u_{r}}{\partial z}\right)\right]\\ & -\frac{2}{3}\frac{\partial }{\partial z}\left\{\eta \left[\frac{\partial u_{z}}{\partial z}+\frac{1}{r}\frac{\partial (ru_{r})}{\partial r}\right]\right\}+\sigma E_{r}B_{\theta }+\rho g \end{array}$$

(3) $$\begin{array}{cc} \frac{\partial }{\partial t}\left(\rho u_{r}\right)+\frac{\partial }{\partial z}\left(\rho u_{r}u_{z}\right)+\frac{1}{r}\frac{\partial }{\partial r}\left(r\rho u_{r}u_{r}\right)= & -\frac{\partial P}{\partial r}+\frac{\partial }{\partial z}\left[\eta \left(\frac{\partial u_{r}}{\partial z}+\frac{\partial u_{z}}{\partial r}\right)\right]+\frac{2}{r}\frac{\partial }{\partial r}\left(r\eta \frac{\partial u_{r}}{\partial r}\right)\\ & -\frac{2}{3}\frac{\partial }{\partial r}\left\{\eta \left[\frac{\partial u_{z}}{\partial z}+\frac{1}{r}\frac{\partial (ru_{r})}{\partial r}\right]\right\}-\eta \frac{2u_{r}}{r^{2}}-\sigma E_{z}B_{\theta } \end{array}$$

(4) $$\begin{array}{cc} \frac{\partial }{\partial t}\left(\rho h\right)+\frac{\partial }{\partial z}\left(\rho hu_{z}\right)+\frac{1}{r}\frac{\partial }{\partial r}\left(r\rho hu_{r}\right)= & \frac{\partial }{\partial z}\left(\frac{\lambda }{C_{P}}\frac{\partial h}{\partial z}\right)+\frac{1}{r}\frac{\partial }{\partial r}\left(r\frac{\lambda }{C_{P}}\frac{\partial h}{\partial r}\right)\\ & +\frac{\partial P}{\partial t}+u_{z}\frac{\partial P}{\partial z}+u_{r}\frac{\partial P}{\partial r}-Q_{rad}+Q_{con}+\Phi +\sigma \left(E_{z}^{2}+E_{r}^{2}\right)+S_{e} \end{array}$$

(5) $$\frac{\partial }{\partial z}\left(\sigma E_{z}\right)+\frac{1}{r}\frac{\partial }{\partial r}\left(r\sigma E_{r}\right)=0,$$

(6) $$\frac{1}{r}\frac{\partial }{\partial r}\left(rB_{\theta }\right)=\mu_{0}\sigma E_{z},$$

where *ρ* represents the density of fluid, *t* denotes time, *u* stands for velocity, *S_m_* is the net production rate of mass from the molten pool surface to the fluid region, *P* denotes pressure, *η* represents viscosity, *σ* denotes electrical conductivity, *E* stands for the electric field, *B* denotes the magnetic flux density, *g* represents gravitational acceleration, *h* denotes enthalpy, *λ* stands for thermal conductivity, *C_P_* is the specific heat at constant pressure, *Q_rad_* represents the radiation loss, *Q_con_* denotes heat generation by condensation, *Φ* stands for viscous dissipation, *S_e_* is the net production rate of energy around the molten pool surface, and *μ*_0_ represents permeability in vacuum. Subscripts *z*, *r*, and *θ* respectively denote the axial, radial, and azimuthal components. Equations (1)–(5) respectively describe conservation of mass, axial momentum, radial momentum, energy, and electric current density. Equation (6) expresses the relation between the electric current and the magnetic field. The momentum exchange at particle generation is negligible. The current balance considering electron emission and ion recombination at the electrode surfaces are treated as described in an earlier report [32].

In engineering temporal and spatial scales, the aerosol dynamics approach describes the growth and transport processes of nanoparticles. Nemchinsky and Shigeta [33] proposed a simple set of equations describing the collective growth of aerosol particles. They demonstrated that it obtained almost identical number density and mean diameter as the MOM with a complex description [34]. By extension of their model [33], a model that also expresses nanoparticle transport by convection and diffusion has been proposed as [18,21,28]

(7) $$\begin{array}{cc} \rho \frac{\partial }{\partial t}\left(\frac{n_{v}}{\rho }\right)+\rho u_{z}\frac{\partial }{\partial z}\left(\frac{n_{v}}{\rho }\right)+\rho u_{r}\frac{\partial }{\partial r}\left(\frac{n_{v}}{\rho }\right)= & \frac{\partial }{\partial z}\left[\rho D_{v}\frac{\partial }{\partial z}\left(\frac{n_{v}}{\rho }\right)\right]+\frac{1}{r}\frac{\partial }{\partial r}\left[r\rho D_{v}\frac{\partial }{\partial r}\left(\frac{n_{v}}{\rho }\right)\right]\\ & -Jq_{c}-\beta_{0}(n_{v}-n_{s})n_{p}^{1/3}f^{2/3}+S_{v} \end{array}$$

(8) $$\begin{array}{cc} \rho \frac{\partial }{\partial t}\left(\frac{n_{p}}{\rho }\right)+\rho u_{z}\frac{\partial }{\partial z}\left(\frac{n_{p}}{\rho }\right)+\rho u_{r}\frac{\partial }{\partial r}\left(\frac{n_{p}}{\rho }\right)= & \frac{\partial }{\partial z}\left[\rho D_{p}\frac{\partial }{\partial z}\left(\frac{n_{p}}{\rho }\right)\right]+\frac{1}{r}\frac{\partial }{\partial r}\left[r\rho D_{p}\frac{\partial }{\partial r}\left(\frac{n_{p}}{\rho }\right)\right]\\ & +J-2\sqrt{2}\beta_{0}n_{p}^{11/6}f^{1/6} \end{array}$$

(9) $$\begin{array}{cc} \rho \frac{\partial }{\partial t}\left(\frac{f}{\rho }\right)+\rho u_{z}\frac{\partial }{\partial z}\left(\frac{f}{\rho }\right)+\rho u_{r}\frac{\partial }{\partial r}\left(\frac{f}{\rho }\right)= & \frac{\partial }{\partial z}\left[\rho D_{p}\frac{\partial }{\partial z}\left(\frac{f}{\rho }\right)\right]+\frac{1}{r}\frac{\partial }{\partial r}\left[r\rho D_{p}\frac{\partial }{\partial r}\left(\frac{f}{\rho }\right)\right]\\ & +Jq_{c}+\beta_{0}(n_{v}-n_{s})n_{p}^{1/3}f^{2/3} \end{array}$$

where *n* represents the number density, *D* is the diffusion coefficient, and *S_v_* is the net production rate of vapor molecules from the molten pool surface to the fluid region. Subscripts *p*, *v*, and *s* respectively denote particle, vapor, and saturated state. Variable *f* is defined as *f* = *n_p_q*, where *q* is the average monomer number in a particle. Equations (7)–(9) respectively describe conservations of vapor molecules, nanoparticles, and total number of monomers in both gas and particle phases at local positions. These equations are written in Eulerian expressions. Therein, it is assumed that the material vapor molecules move with a flow and gradually form nuclei and small nanoparticles that also move with the flow.

*D_p_* is the diffusion coefficient of particles derived from [27] as

(10) $$D_{p}=\frac{k_{B}T}{3\pi \eta d_{v}}\left(q^{-1/3}+3.314\frac{l}{d_{v}}q^{-2/3}\right),$$

where *k_B_* is Boltzmann’s constant, *T* represents the temperature, *d* denotes the diameter, and *l* is the mean free path. In addition, *D_v_* is the diffusion coefficient of material vapor obtained from the second viscosity approximation in the literature [35]. *J* is the homogeneous nucleation rate. *q_c_* represents the number of monomers composing a particle in a critical state, as estimated using the modified self-consistent nucleation theory presented by Girshick et al. [36]. Additionally, *β*_0_ is a parameter related to the collision frequency given as [33]:

(11) $$\beta_{0}={\left(\frac{3v_{v}}{4\pi }\right)}^{1/6}\sqrt{\frac{6k_{B}Tv_{v}}{m_{v}}},$$

where *v* denotes the volume and m represents the mass. The fourth term of the right-hand side of Equation (8) signifies the contribution of coagulation among particles. The fourth terms on the right-hand sides of Equations (7) and (9) signify the contribution of heterogeneous condensation. Therefore, the heat generation because of condensation in Equation (4) is

(12) $$Q_{con}=m_{v}H_{v}\beta_{0}(n_{v}-n_{s})n_{p}^{1/3}f^{2/3},$$

where *H_v_* denotes the latent heat of vaporization. The emission flux of iron vapor molecules from the molten pool surface can be estimated as approximately $\left(P_{s}-P_{v}\right)/\sqrt{2\pi m_{v}k_{B}T}$ from the kinetic theory [37]. This growth–transport model obtains the spatial distributions of the number density and mean diameter of nanoparticles with a lower computational cost than those of other models [12,13,14,15,16,17,19,20,21,22,23,24,25,26,27].

To solve those governing equations, a computational method, “Method-III” proposed in [29], was used. This method not only expresses plasma flow dynamics, it can also capture a particle distribution with steep gradients in and around plasma, which has large variations of density and transport properties. For the 2D axisymmetric domain in Figure 2, a time-dependent simulation was conducted using a Cartesian staggered grid system with a uniform spatial interval of Δ*z* = Δ*r* = 0.1 mm and a time increment of Δ*t* = 0.1 ms. The non-slip condition was imposed at the solid and liquid surfaces. For the downstream outlet boundary, the unsteady outflow condition based on mass conservation considering variable density [38] was adopted. The temperature-dependent thermodynamic and transport properties of argon, which include the effects of ionization in LTE, were obtained from the literature [39]. Temperature-dependent radiation was referred from the literature [40]. The material properties of tungsten and iron were obtained from the database [41].

It is noteworthy that a turbulent flow has an eddy diffusion effect on the temperature field and the vapor and nanoparticle distributions [17,28]. Although the present model accompanies no turbulence model, time-dependent eddy motions larger than the grid scale were expressed. Smaller eddy motions, which turbulence models treat, were neglected. However, the subject of this paper can be discussed even on this simplification. Therefore, the present study excluded the factor of turbulence models to clarify and simplify the problem and discussion.

## 3. Results and Discussion

**Figure 3:**
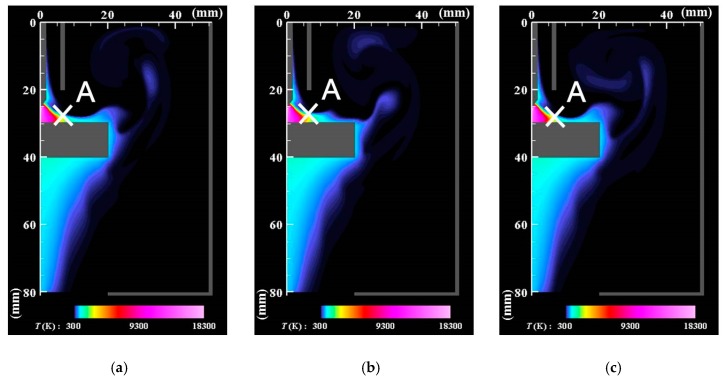
Instantaneous distributions of temperature. (**a**) *t* = *t*_0_; (**b**) *t* = *t*_0_ + 5 ms; (**c**) *t* = *t*_0_ + 10 ms. The arc plasma has a bell-like shape with a maximum temperature of approximately 18,000 K. A recirculating flow is observed above the anode.

**Figure 4:**
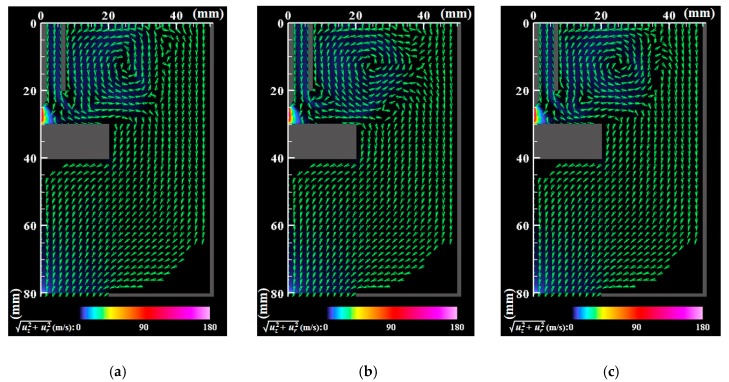
Instantaneous distributions of velocity vectors. Vectors slower than 0.1 m/s are blanked out. (**a**) *t* = *t*_0_; (**b**) *t* = *t*_0_ + 5 ms; (**c**) *t* = *t*_0_ + 10 ms. The arc plasma has a maximum speed of approximately 180 m/s. The outer part of the shielding gas forms a recirculating flow above the anode, merges with the downward flow, and finally flows out of the bottom exit.

Figure 3 and Figure 4 present the instantaneous distributions of temperature and velocity vectors. *t*_0_ denotes a time when the behavior is regarded as quasiperiodic. The present simulation produces a bell-like shape arc plasma with a maximum temperature of approximately 18,000 K, which agrees with the measurement result obtained using spectroscopy [42]. The inner part of the shielding gas flows into the arc plasma, gains thermal energy, flows out of the arc plasma on the base metal surface, and turns downward to the bottom of the domain. The outer part of the shielding gas flows outward, rolls up, merges with the downward flow, and finally flows out of the bottom exit.

**Figure 5:**
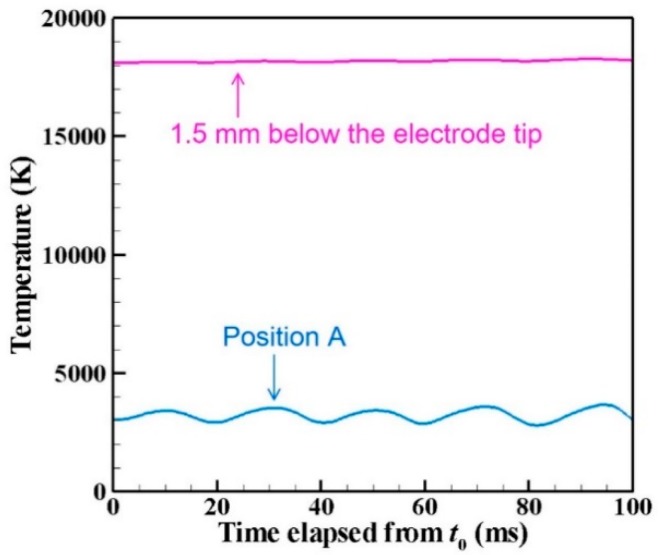
Time evolution of temperatures at two positions. The position below the electrode tip exhibits almost stationary temperature of approximately 18,000 K, whereas position A at the fringe shows fluctuating temperature around 3000 K.

Figure 5 depicts time evolutions of the temperatures at the position of 1.5 mm below the electrode tip (*z*, *r*) = (26.5 mm, 0 mm) and the position at the arc plasma’s fringe (*z*, *r*) = (28 mm, 6 mm) shown as A in the figure. The position below the electrode tip exhibits almost stationary temperature of approximately 18,000 K, whereas the position A at the fringe shows fluctuating temperature around 3000 K because of fluid dynamic instability between the arc plasma and the shielding gas.

**Figure 6:**
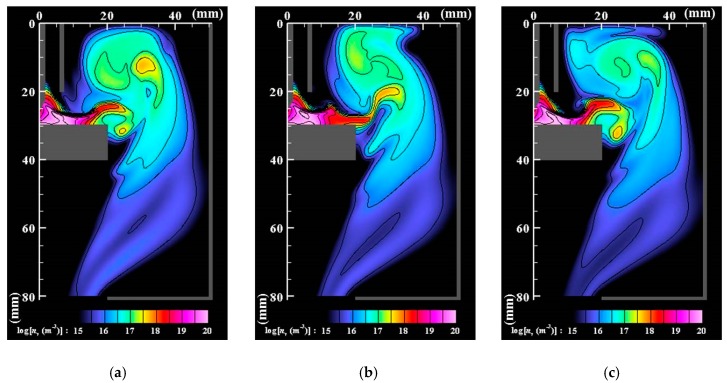
Instantaneous distributions of number density of vapor molecules. (**a**) *t* = *t*_0_; (**b**) *t* = *t*_0_ + 5 ms; (**c**) *t* = *t*_0_ + 10 ms. The vapor molecules are emitted from the molten pool surface and are transported in and around the arc plasma. The number of vapor molecules decreases by condensation in the downstream region.

**Figure 7:**
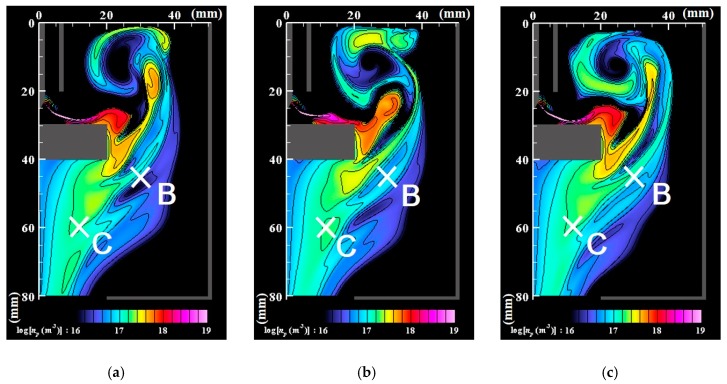
Instantaneous distributions of the number density of nanoparticles. (**a**) *t* = *t*_0_; (**b**) *t* = *t*_0_ + 5 ms; (**c**) *t* = *t*_0_ + 10 ms. At the arc plasma fringe, the vapor molecules change their phase to particles through nucleation, condensation, and coagulation. The nanoparticles decrease in the downstream region because of coagulation.

**Figure 8:**
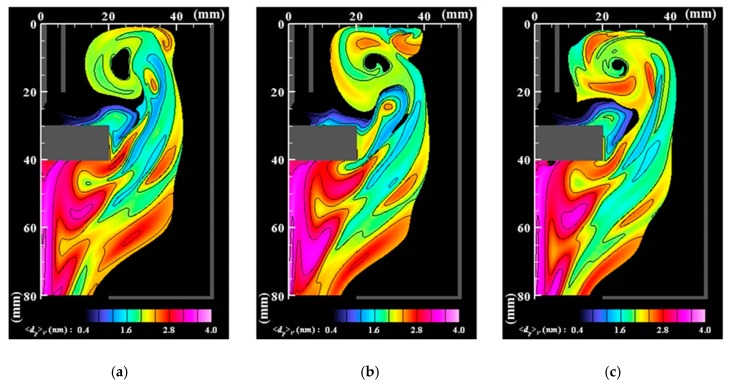
Instantaneous distributions of mean diameters of nanoparticles (cutoff for *n_p_* < 10^16^ m^−3^). (**a**) *t* = *t*_0_; (**b**) *t* = *t*_0_ + 5 ms; (**c**) *t* = *t*_0_ + 10 ms. The nanoparticles have larger sizes in downstream regions because of the growth of condensation and coagulation.

Figure 6, Figure 7 and Figure 8 respectively portray the instantaneous distributions of vapor molecule number density, particle number density, and particle mean diameter at the same moments as Figure 3. The vapor molecules are emitted from the molten pool surface and are transported in and around the arc plasma. Because the vapor molecule temperature decreases at the arc plasma’s fringe, the molecules become supersaturated and change their phase to particles through nucleation, condensation, and coagulation. Therefore, numerous small particles exist at the arc plasma’s fringe. The number of vapor molecules decreases remarkably by condensation in the downstream region. The nanoparticles also decrease in the downstream region. The larger size regions in Figure 8 coincide with smaller number density regions in Figure 7. These results indicate that simultaneous coagulation decreasing the particle number plays a considerably important role in particle growth as well.

The present simulation exhibits mean diameters ranging from subnanometer scale to 4 nm. This size range seems reasonable. Several studies using experimentation have demonstrated that ultrafine particles produced by thermal plasmas have widely dispersed size distributions ranging from a few to a few tens of nanometers, although thermal plasmas of different types were used [1,12,19,43,44,45,46,47]. For instance, Yoshida and Akashi [43] reported that ultrafine iron particles produced using a radiofrequency plasma had mean diameters (statistical medians) smaller than approximately 10 nm. Berlinger et al. [1] fabricated iron-based nanoparticles in several arc plasma discharge conditions. They showed that many particles smaller than a few tens of nanometers were generated. Mean diameters predicted by the present simulations are in those practical ranges. It should be emphasized that particle diameters smaller than a few nanometers usually could not be measured in experiments, even if such small particles are produced.

As shown by earlier studies using experimentation [47] and computation [48], the cooling rate at the fringe of plasma strongly affects the particle growth of nucleation and condensation. The cooling rate is affected by the temperature and flow in and around plasma. They depend on plasma discharge conditions, such as electrical current, voltage, shielding gas, and electrodes. It is noteworthy that the nanoparticle yield and size can be controlled by adjusting those external parameters.

Nanoparticles are generated after the vapor molecules experience supersaturation during the temperature decrease around the arc plasma fringe, at which time, small temperature fluctuations are also generated by fluid dynamic instability. Apparently, the upstream temperature fluctuations affected the downstream particle distribution. To assess this feature, cross-correlation between these two factors is evaluated. Specifically, the cross-correlation coefficients for the temperature fluctuations at a fixed position and of the number density of particles are calculated as

(13) $$R_{cross}(\tau ,z,r)=\frac{\int_{t_{0}}^{t_{1}}T^{\prime }(t,z_{0},r_{0})\cdot {n_{p}}^{\prime }(t+\tau ,z,r)dt}{\sqrt{\int_{t_{0}}^{t_{1}}{\left|T^{\prime }(t,z_{0},r_{0})\right|}^{2}dt}\cdot \sqrt{\int_{t_{0}}^{t_{1}}{\left|{n_{p}}^{\prime }(t,z,r)\right|}^{2}dt}},$$

using quasi-periodic data during 128 ms (=*t*_1_ − *t*_0_). Here, *τ* represents the time lag. The prime mark denotes fluctuation defined as the difference from the time-averaged value. The fixed position (*z*_0_, *r*_0_) = (28 mm, 6 mm) at the arc plasma’s fringe was selected for the temperature fluctuation as anchoring point A, as depicted in Figure 3.

**Figure 9:**
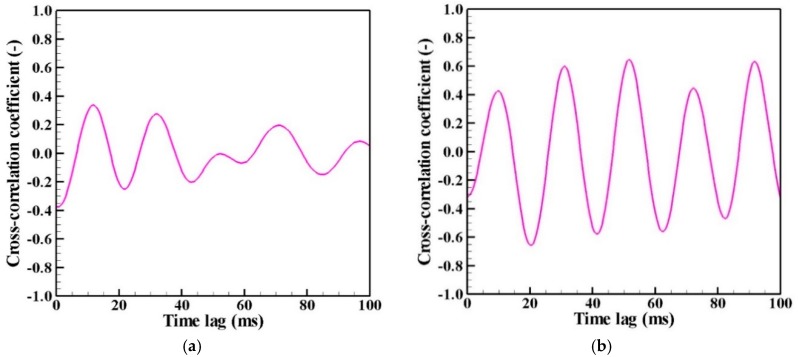
Cross-correlation coefficients: (**a**) between A and B; (**b**) between A and C. Distant position C exhibits stronger correlation than the more proximate position B.

**Figure 10:**
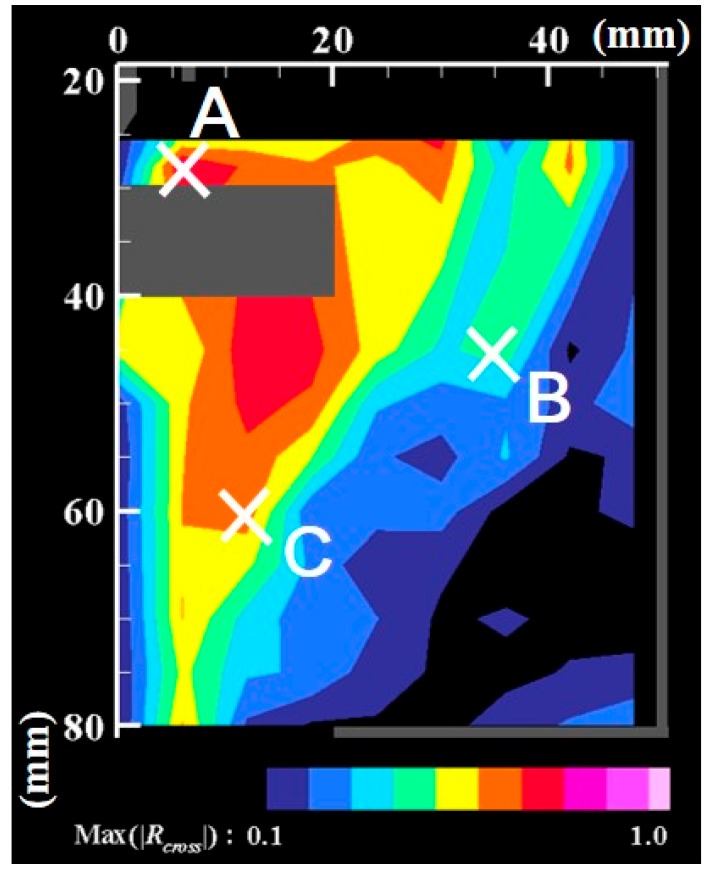
Map of maximum cross-correlation coefficient magnitudes. Temperature fluctuation at the arc plasma’s fringe affects the growth and transport of nanoparticles in a far downstream region.

Figure 9 depicts examples of the cross-correlation coefficients obtained for the fluctuation of the number density of nanoparticles at the positions (*z*, *r*) = B (45 mm, 30 mm) and C (60 mm, 12 mm), as portrayed in Figure 7. Figure 10 presents a map of the maximum values of the cross-correlation coefficient magnitude obtained at each position. It is readily apparent that the dynamic behavior of the nanoparticle distribution is correlated closely with temperature fluctuation at the arc plasma fringe. The closer positions have stronger correlations, which seems natural. It is interesting how the distant position C exhibits stronger correlation than the more proximate position B. This result suggests that the distribution of growing nanoparticles not near but distant from the arc plasma can be controlled via control of the temperature fluctuation at the arc plasma fringe.

## 4. Conclusions

A time-dependent 2D axisymmetric simulation was conducted for arc plasma with dynamically fluctuating fluid generating iron nanoparticles in a direct-current discharge condition. The nonequilibrium process of simultaneous growth and transport of nanoparticles was simulated using a simple model with lower computational cost. To capture fluid dynamic instability and steep gradients in plasma temperature and particle distributions, a highly accurate method was adopted for the computation. The major results revealed by this study are explained below.

➢ The core region of the arc plasma is almost stationary, whereas the fringe fluctuates because of fluid dynamic instability between the arc plasma and the shielding gas.
➢ Numerous small particles are generated around the arc plasma’s fringe because of supersaturation. In the downstream region, the vapor molecules decrease by condensation. The nanoparticles decrease by coagulation. These processes are important contributions to particle growth.
➢ The correlation analysis results suggest that the distribution of growing nanoparticles distant from the arc plasma can be controlled via control of the temperature fluctuation at the arc plasma’s fringe.

## Figures and Tables

**Table 1:** Conditions of arc plasma discharge.

Property	Parameter
Cathode material	Tungsten with 5.0 wt % of La_2_O_3_
Cathode diameter	3.2 mm
Cathode length	25.0 mm
Cathode tip angle	60.0 degrees
Anode material	Iron
Anode diameter	40.0 mm
Anode thickness	10.0 mm
Distance between cathode and anode	5.0 mm
Shielding gas nozzle material	Iron
Shielding gas nozzle inner diameter	12.2 mm
Shielding gas nozzle length	20.0 mm
Shielding gas nozzle thickness	1.0 mm
Shielding gas	Argon
Shielding gas flow rate	15.0 L/min
Current	150.0 A
Voltage	10.5 V
Outer case material	Iron
Outer case inner diameter	100.0 mm
Outer case length	80.0 mm
Outer case outlet diameter	40.0 mm
Outer case temperature	300.0 K
